# Association of sociodemographic and environmental factors with spatial distribution of tuberculosis cases in Gombak, Selangor, Malaysia

**DOI:** 10.1371/journal.pone.0252146

**Published:** 2021-06-17

**Authors:** Nur Adibah Mohidem, Malina Osman, Zailina Hashim, Farrah Melissa Muharam, Saliza Mohd Elias, Rafiza Shaharudin

**Affiliations:** 1 Department of Environmental and Occupational Health, Faculty of Medicine and Health Sciences, Universiti Putra Malaysia, Serdang, Selangor, Malaysia; 2 Department of Medical Microbiology, Faculty of Medicine and Health Sciences, Universiti Putra Malaysia, Serdang, Selangor, Malaysia; 3 Department of Agriculture Technology, Faculty of Agriculture, Universiti Putra Malaysia, Serdang, Selangor, Malaysia; 4 Institute for Medical Research, National Institutes of Health, Shah Alam, Selangor, Malaysia; National Taiwan University, TAIWAN

## Abstract

Tuberculosis (TB) cases have increased drastically over the last two decades and it remains as one of the deadliest infectious diseases in Malaysia. This cross-sectional study aimed to establish the spatial distribution of TB cases and its association with the sociodemographic and environmental factors in the Gombak district. The sociodemographic data of 3325 TB cases such as age, gender, race, nationality, country of origin, educational level, employment status, health care worker status, income status, residency, and smoking status from 1st January 2013 to 31st December 2017 in Gombak district were collected from the *MyTB* web and Tuberculosis Information System (TBIS) database at the Gombak District Health Office and Rawang Health Clinic. Environmental data consisting of air pollution such as air quality index (AQI), carbon monoxide (CO), nitrogen dioxide (NO_2_), sulphur dioxide (SO_2_), and particulate matter 10 (PM_10_,) were obtained from the Department of Environment Malaysia from 1st July 2012 to 31st December 2017; whereas weather data such as rainfall were obtained from the Department of Irrigation and Drainage Malaysia and relative humidity, temperature, wind speed, and atmospheric pressure were obtained from the Malaysia Meteorological Department in the same period. Global Moran’s I, kernel density estimation, Getis-Ord Gi* statistics, and heat maps were applied to identify the spatial pattern of TB cases. Ordinary least squares (OLS) and geographically weighted regression (GWR) models were used to determine the spatial association of sociodemographic and environmental factors with the TB cases. Spatial autocorrelation analysis indicated that the cases was clustered (*p*<0.05) over the five-year period and year 2016 and 2017 while random pattern (*p*>0.05) was observed from year 2013 to 2015. Kernel density estimation identified the high-density regions while Getis-Ord Gi* statistics observed hotspot locations, whereby consistently located in the southwestern part of the study area. This could be attributed to the overcrowding of inmates in the Sungai Buloh prison located there. Sociodemographic factors such as gender, nationality, employment status, health care worker status, income status, residency, and smoking status as well as; environmental factors such as AQI (lag 1), CO (lag 2), NO_2_ (lag 2), SO_2_ (lag 1), PM_10_ (lag 5), rainfall (lag 2), relative humidity (lag 4), temperature (lag 2), wind speed (lag 4), and atmospheric pressure (lag 6) were associated with TB cases (*p*<0.05). The GWR model based on the environmental factors i.e. GWR2 was the best model to determine the spatial distribution of TB cases based on the highest R^2^ value i.e. 0.98. The maps of estimated local coefficients in GWR models confirmed that the effects of sociodemographic and environmental factors on TB cases spatially varied. This study highlighted the importance of spatial analysis to identify areas with a high TB burden based on its associated factors, which further helps in improving targeted surveillance.

## Introduction

Tuberculosis (TB) is an airborne infectious disease caused by the *Mycobacterium tuberculosis* complex, a type of bacilli that mainly attacks the lungs (pulmonary TB) and other parts of the body (extrapulmonary TB). TB remains one of the top ten causes of mortality worldwide. In 2018, an estimated 1.5 million deaths and 10 millions new TB cases were reported globally, of which 5.7 million (57%) were men, 3.2 million (32%) were women and 1.1 million (11%) were children. Southeast Asian countries accounted for one-third (44%) of the world’s TB burden. In terms of TB incidence, Malaysia ranks 76^th^ worldwide and is classified as a medium-to-low level endemic country [1]. The disease declined by 1.26% from 26,168 cases to 25,837 cases in 2018, most likely due to the directly observed treatment and short-course chemotherapy (DOTS) [2]. Even though Malaysia is not one of the top 30 high TB burden countries in the WHO list, the death rate due to TB is the highest in Malaysia compared to other infectious diseases, i.e. 5–7 deaths per 100 000 population annually [3]. This is worrying because it shows that TB transmission is still active in Malaysia.

Conventionally, it has been proven that sociodemographic factors such as age [4], gender [5], race [6], educational level [7], employment [8], health care worker [9], income [10], residency [11], nationality [12], country of origin [13], and smoking status [14] have a role in TB cases. However, evidence described in the literature confirmed the importance of conducting a study to better understand how air quality and weather can influence the cases of TB. Environmental factors such as air quality index [15], concentration of carbon monoxide [16], nitrogen dioxide [17] and sulphur dioxide [18], particulate matter [19] and weather factors such as rainfall [20], humidity [21], temperature [22], wind speed [23], and atmospheric pressure [24], can enhance the reproduction and growth of *Mycobacterium tuberculosis* to a certain extent.

The mucous lining of the human nasal and respiratory tract surface is the first line of defence to fight against *Mycobacterium tuberculosis* infection by stopping it from entering the pulmonary alveoli [25]. In the early phase of infection, the alveolar macrophages can inhibit the proliferation of *Mycobacterium tuberculosis* through phagocytosis and granuloma formation in the lungs [26]. Exposure to ambient air pollutants reduces the body’s immunity, weakens the clearance of respiratory secretions, makes *Mycobacterium tuberculosis* more susceptible to erosion of alveolar macrophages, and consequently increases the possibility of TB infections [27–29]. Weather is another important factor that could affect the TB cases, principally because air quality is regulated by weather [30]. Different weather indicators may impact the TB cases differently due to the complex interaction of time and space. Biological research generally suggested that change in weather predominantly affects the cases of respiratory diseases such as TB by regulating the distribution and external latency of pathogens and affecting human immunity and pathogen transmission [31]. To further improve the Malaysian TB control strategy, we should pay attention to new or previously neglected risk factors, such as air pollution and weather. However, previous studies [32, 33] did not include the sociodemographic and environmental factors together. Consequently, it is vital to explore the role of spatial heterogeneities in the relationship between risk factors and TB cases in Malaysia, including sociodemographic and environment factors to develop TB control and prevention policies.

Understanding the spatial distribution of a disease is crucial in improving interventions and guiding resource allocation in disease management. Many spatial statistic methods such as spatial autocorrelation analysis (Moran’s I), hotspot analysis (Getis-Ord Gi*), and space-time scan statistic (SaTScan) methods have been used to assess the spatial pattern of TB [34, 35] and other infectious diseases [35–37]. However, these methods were limited to the assessment of spatial variation and they were unable to show the potential factors that influence the models. Most traditional regression analysis such as simple and multiple logistic regression [38–40] is unable to determine the relationship between each independent variable and the dependent variable in space. Although Bayesian methods is able to perform the spatial model, there is a limited way to generalize the uncertainty within background knowledge and prior probability function and it assumes that there is only one true fixed parameter value, even the importance of co-variates in the population [41–43].

In comparison with traditional regression models, spatial regression models have wider advantages especially for samples with completely independent, normally distributed, and random data. The magnitude of the residuals from a regression equation is one of the measures of model fit. Large residuals indicate poor model fit and vice versa. Therefore, the residuals from OLS and GWR model are assumed to be independently and drawn identically from a normally distribution with a mean of zero. It is also assumed to be homoscedastic, in which any samples taken at random from the residuals will have the same mean and variance. Hence, it is more appropriate to be used for data with spatial characteristics. On the other hand, ordinary least squares (OLS) is an approach to estimate the coefficients in a linear regression model to minimize the sum of squared errors of the differences between the actual and predicted value of the outcome variable [44]. Conversely, the geographically weighted regression (GWR) is an alternative approach that allowed the exploration of local patterns in a set of spatial data. However, the limitation of GWR lies within its sensitivity to a small sample size that can result in collinearity, making local coefficients estimates seem counter-intuitive [45]. Furthermore, the bias in GWR may arise from the selection of user-defined bandwidth that determines the level of influence by the neighbouring observations of the local value. Despite these limitations, GWR is still considered as the best method to provide more detailed information for the exploration of spatial variation (non-stationarity). In fact, the GWR model can create an equation for every feature such as each TB case in this study before calibrating it with nearby features. The closer features would have a higher impact on calibration than faraway features. As each feature has it’s equation, coefficients are allowed to vary across space [46]. In short, GWR analysis has the advantage of being able to explain the associated factors of spatial heterogeneity of the disease, especially those that require more attention and prioritisation.

In Malaysia, most of the studies focused on the molecular epidemiology of TB [47–49]. There is a lack of research on the spatial epidemiology of TB. Currently, there are no related studies that analyse on spatial variation of TB in Malaysia. Therefore, this study aimed to determine (i) the trend of TB cases; (ii) the spatial pattern of TB cases; and (iii) the association of sociodemographic and environmental risk factors with TB cases in Gombak from 2013 to 2017.

## Materials and methods

### 1. Study area

**Fig 1:**
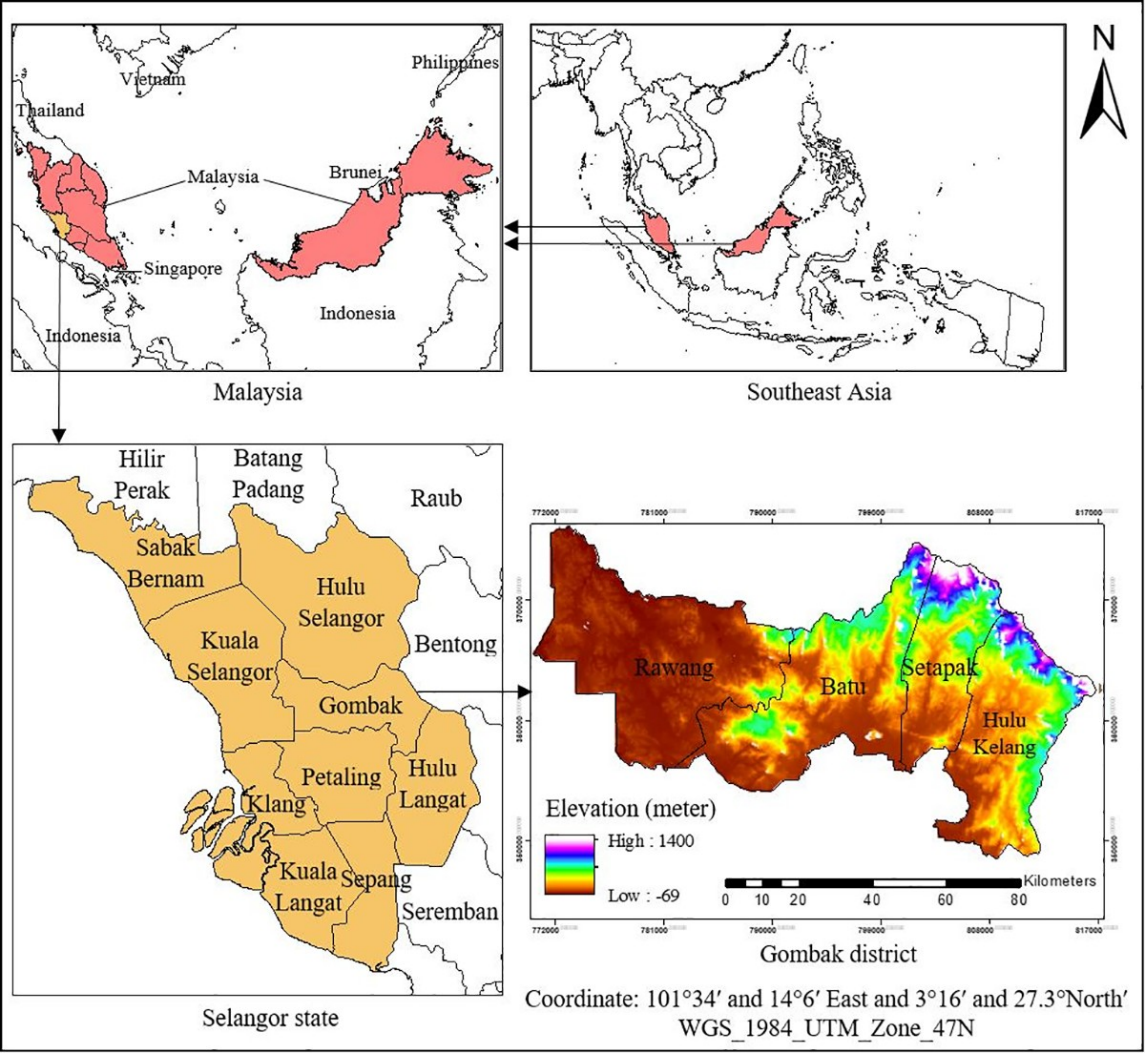
Geographical location of Gombak in the states of Selangor and *mukims* of Gombak.

This cross-sectional study used retrospective data of TB cases in the district of Gombak. Gombak is one of the administrative districts in Selangor with a land area of 650.08 km^2^. The geographic location is between longitude 101°34′ and 14°6′ East and latitude 3°16′ and 27.3°North′ which lies from the middle to the eastern part of Selangor state. Gombak is divided into four sub-districts or *mukim*: Rawang, Batu, Setapak and Hulu Kelang. Each *mukim* is defined as the minimal area of analysis in this study. The location of *mukims* is displayed in Fig 1. Gombak’s residential population is about 815,200 in 2018 which ranks fourth (after Petaling, Hulu Langat, and Klang) among the eight districts in Selangor; approximately 90% of the population lives in urban areas [50]. The annual average temperature is 27.1°C, reflecting the tropical climate which is hot and humid throughout the year. There is significant average rainfall in Gombak, which is about 2535 mm per year [51]. The terrain is hilly in the eastern and parts of the northern and west regions whereby most of them are still covered with forests in the range of altitude between 100 and 500 m above sea level. In the central and south-west, the areas are relatively low with lowlands in the range of average elevation between 30 and 70 m above sea level [52]. Gombak is a part of the Klang Valley zone, which is the area of major municipal areas, especially in the south and west where the rapid urbanization process in the city of Kuala Lumpur spreads to the southern part of Gombak. The district has undergone rapid developmental changes in recent decades, especially those involving industrial processes. The health sector is distributed into two government hospitals, eighteen government clinics, three private hospitals, and three private clinics [53]. In 2018, Selangor accounted for the highest TB burden with 5,071 cases, among which Gombak was the district that reported the highest TB incidence with about 700 cases each year [54].

### 2. Data sources

#### 2.1. Sociodemographic data of TB cases

It is mandatory for all health care providers in hospitals, clinics, institutions of disease prevention and control, and all other designated health care establishments to report active TB cases in a timely manner into the TBIS database of the respective health districts. After that, the data would be electronically transferred into the *MyTB* by Ministry of Health Malaysia’s officers. TB data from 1st January 2013 to 31st December 2014 was collected from Rawang Health Clinic in Rawang while TB data from 1st January 2015 to 31st December 2017 was collected from the TB/Leprosy Unit of Gombak District Health Office in Batu Caves. The data were retrieved via *MyTB* and double checked from TBIS to prevent errors. The data were confidential and can only be accessed with approval from the Director of Gombak District Health Office.

TB is a notifiable infectious disease in Malaysia. According to the Malaysian Practice Guideline on Management of Tuberculosis [55], TB diagnosis is classified as follows: (i) smear positive involves patients diagnosed with at least one sputum smear examination positive for acid fast bacilli (ACB), radiographic abnormalities that are consistent with active TB and/or sputum culture positive for *Mycobacterium tuberculosis*; (ii) smear positive involves patients diagnosed with at least three sputum smear examination negative for ACB, radiographic abnormalities that were consistent with active TB, and/or whose initial sputum smear were negative, who had sputum culture sent for culture initially, and whose subsequent sputum culture result is positive for *Mycobacterium tuberculosis*; (iii) extrapulmonary TB involves organs other than the lung parenchyma, in which diagnosis is based on at least one culture positive specimen from an extrapulmonary site; (iv) drug-resistant TB involves conditions when the *Mycobacterium tuberculosis* is resistant to more than one anti-TB drug such as isoniazid and rifampin, in which the diagnosis can be confirmed by culture, sensitivity test or molecular methods. The TB patients were diagnosed based on X-ray findings, pathogen detection, and pathological diagnosis according to the diagnosis criteria set by the Ministry of Health Malaysia in 2008. Therefore, the case definition of study is defined as the new TB cases being diagnosed during the year regardless the duration of prescribed treatment.

The sociodemographic variables include age (years <15, 15–64, 64>), gender (male, female), race (Malay, Chinese, Indian, others), country of origin (Malaysia, Indonesia, Myanmar, Bangladesh, others), nationality (Malaysian, non-Malaysian), educational level (secondary school and below, higher than secondary school), employment status (employed, unemployed), health care worker status (health care worker and non-health care worker), residency (urban, rural), income status (permanent income, not permanent income), and smoking status (smoking, not smoking). All these data were retrieved via *MyTB* and double checked from TBIS to prevent errors. These variables were selected through the consideration of the real situation in Malaysia from the references of previous literatures [56, 57]. Other data such as individual’s ID, type of TB (smear positive, smear negative, extrapulmonary, drug-resistant), and date of diagnosis (month, year) were also collected. Additionally, “year” and “month” was defined according to the date when the TB diagnosis was confirmed.

Out of 3,590 notified TB cases, 181 cases (5.04%) were excluded; in which 2.53% were residents outside the study zone while another 2.51% were not diagnosed within the study period. After further removing cases that could not be geocoded (2.46%) due to incorrect, missing, or unclear address information, only 3325 (97.54%) cases were included in the final analysis i.e. 3225 number of TB cases.

#### 2.2. Population data

The total annual population for each *mukim* from 2013 to 2017 was obtained from the Department of Statistic Malaysia.

#### 2.3. Environmental data

**Fig 2:**
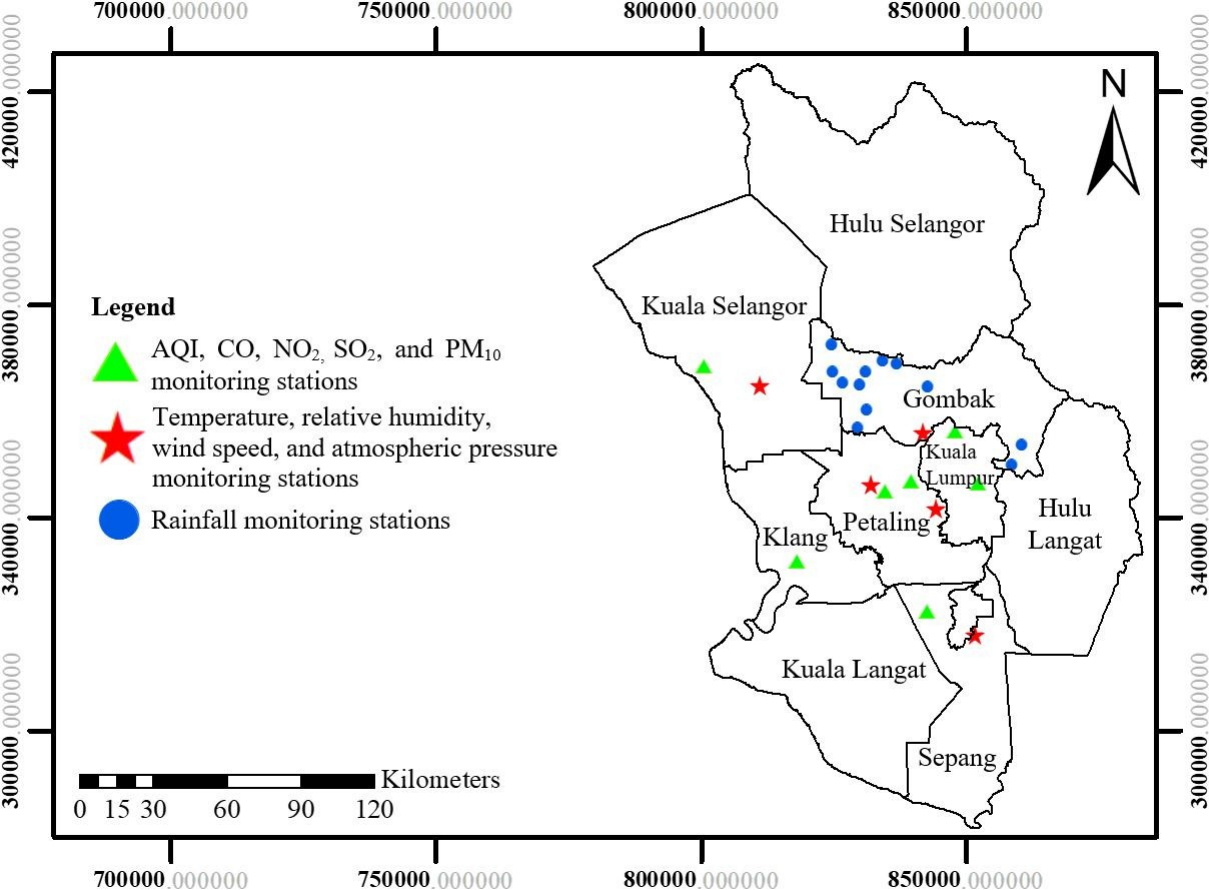
Spatial distribution maps of environmental monitoring stations in Selangor and Kuala Lumpur.

Air pollution data such as air quality index (AQI) and the concentrations (*μ*/m^3^) of carbon monoxide (CO), nitrogen dioxide (NO_2_), and sulphur dioxide (SO_2_), and particulate matter 10 (PM_10_), were obtained from the Department of Environment Malaysia that has seven monitoring stations in Kelang, Petaling Jaya, Shah Alam, Kuala Selangor, Banting, Cheras, and Kuala Lumpur. Rainfall (mm) data were collected from the Department of Irrigation and Drainage Malaysia that has 12 monitoring stations (Batu Arang, Bukit Antarabangsa, Bandar Tasik Puteri, Country Home, Jalan Gombak, Kampung Merbau, Kampung Setia Kuang, Taman Bukit Rawang, Taman Desa Kundang, Taman Garing Utama, Taman Templer, dan Ampang). In addition, other weather data including average humidity (%), average temperature (°C), average wind speed (m/s), and atmospheric pressure (hPa) were obtained from the Malaysian Meteorological Department based on data from the five monitoring stations in Kepong, Sepang, Petaling Jaya, Subang, and Sungai Buloh. All these stations (Fig 2) in Selangor and Kuala Lumpur recorded the level of daily environmental measures from July 2012 to December 2017 either automatically or manually. The data from all these stations were tabulated to produce the monthly average data during the study period. Some studies reported that the estimation of the average incubation period of TB infection ranges from 1–2 months, with a 2-month interval from the symptom appearance to diagnostic test [25, 58]. In addition, reporting of TB to a health care institution also require times that can cause a delay [59]. Leung et al. [60] recommended that the maximum lag time was 6 months. This study set the air pollution and weather factors from 0 to 6 month lag from the diagnosis of TB, which was rational and could be applied to monitor a relatively long-term effect caused by air pollution and weather factors.

Based on the air quality and weather monitoring stations in Fig 2, only few stations are distributed over the area study. The model specification adopted in this study did not allow for any missing values in the variables. Hence, any missing values for the variables were imputed by interpolation from the nearest stations [61]. In this study, kriging was used to interpolate the value of a variable at unsampled locations based on the measurement at nearby locations by fitting a semivariogram model which is a function of spatial distance. Kriging is a proven method to interpolate since it is able to produce the lower errors and better predictions accuracy compared to other geostatistical interpolation methods such as Inverse Distance Weighting and Kernel Smoothing. Further, the fitted model in kriging does not only depend on the distance between the measured points and the prediction location but also the spatial relationships among the measured values around the prediction location [62–64]. Although with datasets consist of relatively few samples, kriging could produce low error estimations [65]. The assessment of the prediction using kriging was conducted to validate the interpolations [66] whereby this study found that the prediction using kriging has very low value of the root mean square error (<0.157) for interpolating air pollution and weather variables.

The data from all the monitoring stations were tabulated to produce the monthly average data during the study period. Different output cell sizes of environmental data were tested (2000m, 1000m, 500m, 200m, and 100m) using the kernel density. Then, Global Moran’s I was used to determine the appropriate output cell size for the interpolation of environmental data in kriging. The results of Global Moran’s I found that 100m yielded the best output. Hence, this study specifically set the cell size of 100m for the output raster dataset in the environment of ArcGIS® version 10.7 (Environmental Systems Research Institute, Inc. Redlands, California, USA). Subsequently, maps were produced in the format of raster files in ArcGIS.

#### 2.4. Geographical data

A polygon shapefile with each *mukim*’s boundary under the administration of Gombak District with a scale of 1:4,000,000 was obtained from the Ministry of Agriculture and Agro-Based Industries, Malaysia. To display the spatial distribution of TB cases and to perform the spatial analysis, the TB data, population data and environmental data were imported into the attribute table of the spatial district data. The Universal Transverse Mercator coordinates of the patient’s residence were geocoded from the address recorded in the database using Google Earth™ version 7.15 (Alphabet Inc., Mountain View, CA, USA). The address includes the location of the patients’ home and institutional settings such as prison, nursing homes, hostels, and homeless shelters. The extracted data were georeferenced with the district polygons in ArcGIS. The spatial unit to conduct the spatial analysis is point data.

#### 2.5. Topographic data

Altitude data showing topographic variation were derived from the 30 m digital elevation model obtained from the Shuttle Radar Topography Mission provided by the United States Geological Survey Earth Explorer.

### 3. Data analysis

#### i. Trend of TB cases

*Incidence rate*. The incidence rate (IR) was used to represent the disease risk across Gombak and to identify the mukims with higher or lower disease risks. The IR was expressed by taking the annual reported TB cases per 100,000 population using the total population of the corresponding mukim as the standard.

The rate was calculated as follows:

$${ir}_{j}=\frac{O_{i}}{N_{i}}x100,000$$ (1)

where *ir*_*j*_ is the incidence rate, *O*_*i*_ is the number of reported TB cases, and *N*_*i*_ is the population in the *i*^*th*^ *mukim* per year. *Mukim* is the smallest administrative area with a reported number of population in Malaysia. Therefore, the IR of TB in this study was based on the *mukim* level only.

*Heat map*. Heatmapping was applied to examine the temporal pattern of TB cases. It presented the monthly variation in the TB cases by mukims and years. This indication could quantify and visualise the periodicity of the disease after adjusting for the sampling differences in the four mukims over the five years by segregating the annual cases of each mukim before using the average annual cases of all mukim. “Month” was defined as the month of the year (January—December). The statistical analysis was performed with Microsoft Excel version 2013 for Windows (Rel. V49O470; Chicago, IL, USA).

#### ii. Spatial pattern of TB cases

*Spatial autocorrelation*. The presence of spatial autocorrelation of TB cases at a global scale was explored using Global Moran’s I to assess the presence, strength, and direction of spatial autocorrelation over the whole study area and to test the assumption of spatial independence in the implementation of the spatial pattern analysis. The scale of analysis was specified as the distance at which the Moran’s I generated a first Z-score peak, an indicator of pronounced spatial aggregation. The initial distance of Moran’s I was 100 meters, a distance at which each feature would has at least one neighbour.

The Global Moran’s *I* index is:

$$I=\frac{n}{S_{0}}+\frac{\sum_{i=1}^{n}{\sum_{j=1}^{n}w_{i,j}Z_{i}Z}_{j}}{\sum_{i=1}^{n}Z_{i}^{2}}$$ (2)

where *Z*_*i*_ = (*x*_*i*_−*X*), *w*_*i*.*j*_ is the spatial weight between feature *i* and *j*, and *n* is the total number of features.


$$S_{0}=\sum_{i=1}^{n}\sum_{j=1}^{n}w_{i,j}$$ (3)

*Z* statistic is the standardisation of Morans’s *I*:

$$Z=\frac{I-E(1)}{\sqrt{V(1)}}$$ (4)

*Kernel density estimation*. Conceptually, kernel density is used to identify certain areas that are riskier than others. In other words, it corresponds with the risk level of point patterns of the TB cases. This technique generalises the case locations to the whole study area which generate a density surface and as an indicative of cluster formation and possible spatial dependence. This continuous space and statistical smoothing technique allow for the filtering of the variability in the data set while maintaining the essential characteristics of the data locations [67]. A symmetrical smooth curved surface over each point in the dataset is fitted using the kernel function to calculate the density of TB cases per unit area. The output cell size of 100m was obtained from the existing cell size value of raster dataset of the environment setting in ArcGIS. The surface value is highest at the location of the point and diminishes with an increasing distance from the point, reaching zero at the search radius distance from the point [68]. The map was generated using a gaussian kernel function on the ArcGIS. The thematic map built a density surface for the visual identification of the spreading pattern of the highest numbers of TB cases. Based on the formula presented below, we attempted to interpolate the dissemination pattern of TB:

$$f(x,y)=\frac{1}{{nh}^{2}}\sum_{i=1}^{n}k(\frac{d_{i}}{h})$$ (5)

where *f*(*x*,*y*) is the density value at location (*x*,*y*), *n* is the number of TB case, *h* is the bandwidth, *d*_*i*_ is the geographical distance between TB case *i* and location (*x*,*y*), and *k* is a density function, known as the kernel.

*Hotspot analysis*. Getis-Ord Gi* statistics was employed to verify the extent to which a location is surrounded by a cluster of hotspot or coldspot of TB cases and also to determine the level of statistical significance for each cluster. A feature has a value; in terms of TB cases, the features are aggregated and their count within the aggregation area represents the value. Significant hotspots would imply a feature with a high value, and they are surrounded by other features with high value as well, whereas significant coldspots would indicate a feature with a low value, and they are surrounded by other features, also with a low value. Gi* statistics is the ratio of the local sum of a feature and its neighbours in the vicinity of a distance or the analysis scale to the sum of all features. When the local sum is very different from the expected local sum, and that difference is too large to be the result of random choice, a statistically significant z-score results.

The significance of clusters at 90%, 95%, or 99% confidence levels corresponds to the value of z-score test of ±1.56, ±1.96, and ±2.58, respectively. The cluster is considered as a hotspot when the value of z-score ≥ +1.56, while the z-score ≤ -1.56 for a coldspot, whereas there is no significance if the z-score value > -1.56 and < +1.56. For statistically significant positive z-score, the higher z-score values indicate more intense clustering of hotspot. On the other hand, for statistically significant negative z-score, the lower z-score values indicate more intense clustering of coldspot. The statistically significant hotspot and coldspot from the results of z-score test are represented through the Gi-Bin values which +3 and -3 reflects a 99% confidence level, +2 and -2 reflects a 95% confidence level, and +1 and -1 reflects a 90% confidence level, with zero reflects not being statistically significant. Similar with the z-score values, higher confidence levels presume a more likely aggregation of hotspot or coldspot. Detection of cases with more than 90% higher confidence levels is considered hotspot or coldspots with relation to the hypothesis of spatial randomness.

#### iii. Regression analysis

*Ordinary least square*. The ordinary least square (OLS) is a global model and expects the variable relationship to be constant (stationary) throughout the study area. The fundamental assumption of a multivariate regression model is that the relation of dependent and independent (explanatory) variable is spatially constant [69]. Even though the OLS model is not the best method for the analysis of spatial data, it has consistently been the proper starting point for all spatial regression analyses to screen the significant indicators (explanatory variable) associated with TB cases (dependent variable).

In the first step, this study conducted OLS regression to test the assumption of the models according to OLS requirements: (i) coefficients for model explanatory variables should be statistically significant and have the expected sign (+/-), (ii) probability for model explanatory variables should less than 0.05, (iii) explanatory variables should be free from multicollinearity ([Variance Inflation Factor (VIF)] value is <7.5), (iv) residuals must be normally distributed with a mean of zero, (v) residuals must not be spatially autocorrelated and (vi) the model should not be biased (heteroscedasticity or non-stationarity) [70]. The spatial independence of residuals was evaluated using the spatial autocorrelation coefficient, Global Moran’s I index. An OLS model can be described with the following equation:

$$Y_{i}={TB}_{i}=\beta_{0}+\beta_{0}X_{i}+\beta_{0}X_{i}+\cdots \ldots \ldots \ldots \ldots ..+\varepsilon_{j}$$ (6)

where *Y*_*i*_ is the dependent variable (TB cases) measured at some location *i*, *X*_*i*_ is the explanatory variable, *ε*_*j*_ is a random error term assumed to be normally distributed, *β*_0_ is the intercept and *β*_*i*_ is the explanatory variables to be estimated.

Three models were tested as follows (i) OLS1: gender, nationality, residency, educational level, employment status, health care worker status, income status, and smoking status; (ii) OLS2: environmental variables: AQI (lags 1–3), CO (lags 2–4), NO_2_ (lags 2 and 3), SO2 (lags 1–6), PM_10_ (lags 5 and 6), rainfall (lag 2–6), temperature (lags 1–5), wind speed (lags 4 and 5), relative humidity (lags 2–6), and atmospheric pressure (lag 6), and (iii) OLS3: gender, nationality, residency, employment status, income status, AQI (lags 1–3), CO (lags 2–4), NO_2_ (lags 2 and 3), SO_2_ (lags 1–6), PM_10_ (lags 5 and 6), rainfall (lag 2–6), temperature (lags 1–5), wind speed (lags 4 and 5), relative humidity (lags 2–6), and atmospheric pressure (lag 6).

*Geographically weighted regression*. In the OLS analysis, the association between risk factors and disease is unlikely to be stationary and more likely to vary over space. The parameter estimates might thus demonstrate significant spatial variations [71]. GWR is an improvement of traditional regression models whereby it extends and modifies the global regression technique by enabling the estimation of local parameters rather than global statistics, thereby making it possible to address the spatial variations among variables. Hence, the geographically weighted regression (GWR) models were developed to examine the presence of spatial non-stationarity in the association between variables. The meaning for the assumption of spatial non-stationarity is the correlation between the explanatory and dependent variables are not the same for the entire area [72]. Therefore, the GWR model was able to explore the variation in the relationship between cases and risk factors geographically. This process allows an evaluation of the spatial heterogeneity in the estimated associations between TB cases and the sociodemographic and environmental factors. The local parameters change into the spatial positions of the data and the standard errors of the coefficients to illustrate the reliability of the estimated coefficients [69]. GWR is a local regression model that creates an equation for every feature (every TB case in this context) to calibrate it with nearby features. The closer features have a larger impact on the calibration compared to faraway features. As each feature has its equation, the coefficients would be varied over space [72]. To build the model, the critical part lies in the allocation of optimal bandwidth and the best value to be chosen. Considering that the samples were not regularly spaced in the Gombak district, various combinations of adaptive bandwidth and gaussian kernel were implemented to choose the most suitable model. A GWR model can be described with the following equation:

$$y_{j}=\beta_{0}(\mu_{j},v_{j})+\sum_{i=1}^{p}\beta_{i}(\mu_{j},v_{j})X_{ij}+\varepsilon_{j}$$ (7)

where (μ_*j*_,*v*_*j*_*)* is the spatial coordinate of sample point *j*, *β*_0_(μ_*j*_, *v*_*j*_) and *β*_*i*_(*μ*_*j*_, *μ*_*j*_) are the regression constant and the regression coefficient of sample point *j*, and *ε*_*j*_ is the random error of the independent distribution.

The AIC that was fixed by the maximum likelihood principle was also used to determine the optimal bandwidth. With the use of GWR, three models were analysed using a similar factor of TB cases with the OLS analysis but under different variables as follows: (i) GWR1: gender, nationality, employment status, health care worker status, income status, residency, and smoking status; (ii) GWR2: AQI (lag 1), CO (lag 2), NO_2_ (lag 2), SO_2_ (lag 1), PM_10_ (lag 5), rainfall (lag 2), relative humidity (lag 4), temperature (lag 2), wind speed (lag 4), and atmospheric pressure (lag 6); and (iii) GWR3: nationality, income status, residency, CO (lag 2), NO_2_ (lag 2), SO_2_ (lag 1), PM_10_ (lag 5), rainfall (lag 2), temperature (lag 2), and atmospheric pressure (lag 6). The bandwidth size was selected through the golden-section search, in which a bandwidth size of 25 was used to build GWR1, whereas 34 and 30 were used to build GWR2 and GWR3, respectively. R^2^, adjusted R^2^, and AIC values were used to compare the fitness of the OLS and GWR models [73]. Variance inflation factors (VIFs) were applied to monitor multicollinearity among the indicators [74]. The R^2^ and adjusted R^2^ were chosen to compare the overall fitness of each GWR model while local R^2^ was selected to explore the spatial variability of combinatory set of explanatory variables in GWR1, GWR2, and GWR3 at each location of TB cases in Gombak. Specifically, distribution of the local R^2^ values in the form of map could display the level of association between each combinatory set of explanatory variables with TB cases across the district.

### 4. Ethical considerations

The use of secondary data in this study was approved by the Medical Review & Ethics Committee, Ministry of Health Malaysia and registered under the National Medical Research Registry (NMRR-17-3029-39236).

## Results

### i. Trend of TB cases

**Fig 3:**
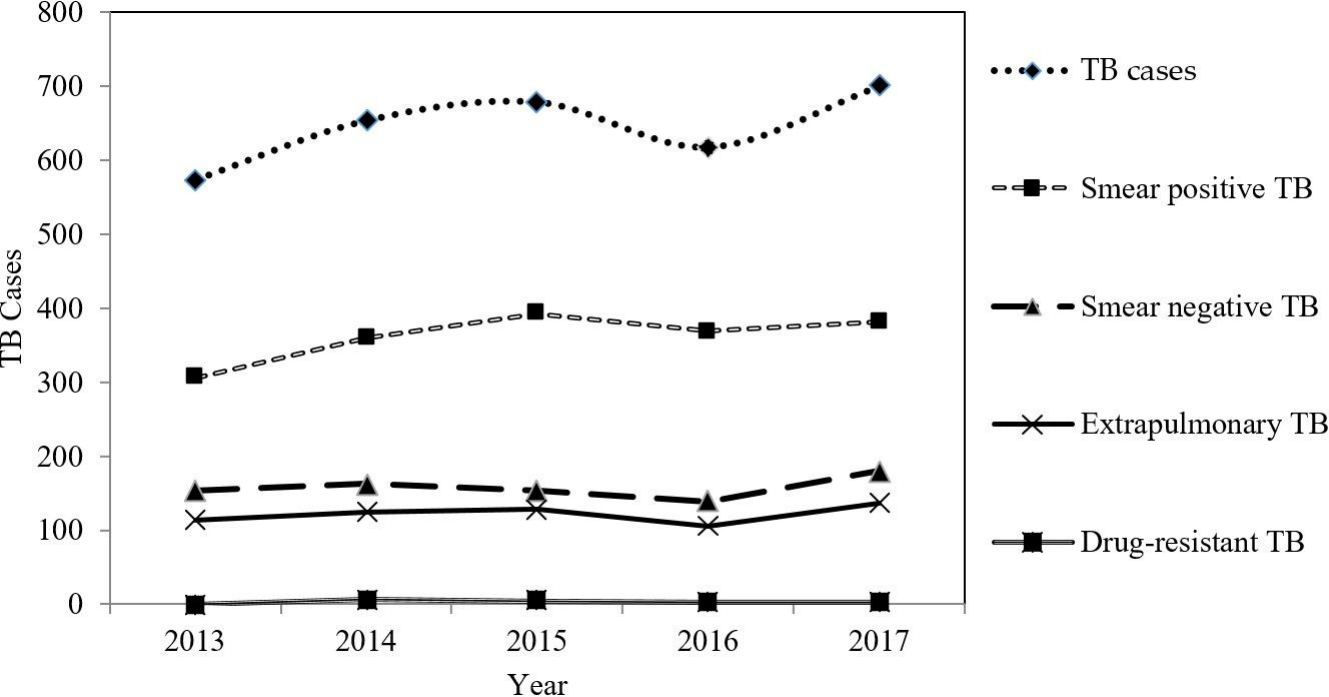
The trend of TB cases in Gombak, 2013 to 2017.

The annualised average cases of smear positive TB, smear negative TB, extrapulmonary TB, drug-resistant TB, and total TB cases over the five-year period were 362.20, 158.00, 121.80, 3.00, and 645.00 cases respectively. From 2013 to 2017, the trend of smear positive TB was relatively stable and only increased slightly from 306 to 382 cases. The slow rising trend of TB cases from 2013 to 2015 was mainly caused by the increased number of smear positive TB cases while the slow decreasing trend from 2015 to 2016 was due to the reduction in the smear positive TB, smear negative TB, and extrapulmonary TB cases in combination. On the other hand, the slightly increasing trend from 2016 to 2017 was due to the increment in the smear negative TB and extrapulmonary TB (Fig 3).

**Fig 4:**
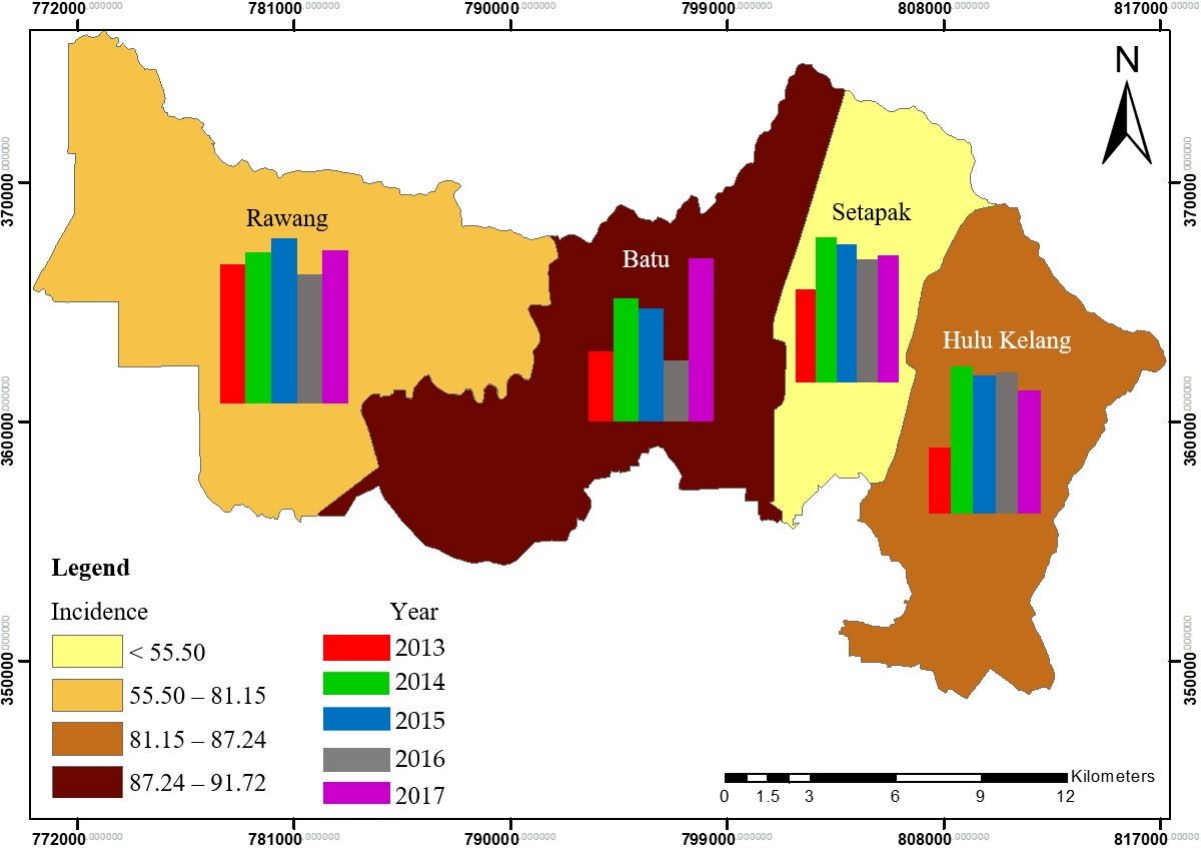
Average incidence rate of TB from 2013 to 2017 at the *mukim* level in Gombak.

The spatial distribution of incidence rate (IR) for each *mukim* in Gombak over the five-year period is presented in Fig 4. The colour of each *mukim* indicated the average IR of TB and the bar charts represented the annual IR from 2013 to 2017 for each *mukim*. Four group of IRs were formed using the natural break method by clustering similar range of values and maximising the gap of each group. The highest average IR of TB was in Batu (91.72 cases per 100,000 population), followed by Hulu Kelang (87.24 cases per 100,000 population). The lowest average IR of TB was in Setapak (55.49 cases per 100,000 population). The average IR of TB for each *mukim* from 2013 to 2014 (Fig 3) showed a slightly increasing trend for each *mukim*. IR in 2016 in all *mukim*s dipped and then increased again in 2017 except for Hulu Kelang. The highest IR was in 2015 for Rawang, 2017 for Batu, and 2014 for Setapak and Hulu Kelang, whereas the lowest IR was in 2016 for Rawang and Batu, while 2013 for Setapak and Hulu Kelang. For the whole Gombak, the highest IR was in 2015 (83.44 cases per 100,000 population) while the lowest IR was in 2013 (71.16 per 100,000 population). For the average of the five-year period, Batu had the highest IR (91.72 cases per 100,000 population) while Setapak had the lowest IR (55.49 cases per 100,000 population).

**Fig 5:**
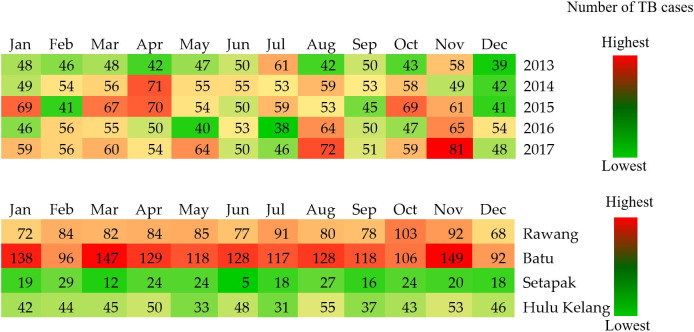
Heat map of TB cases by year and by *mukim* in Gombak, 2013 to 2017.

The temporal pattern of TB cases by year and *mukim* in Gombak is illustrated in the heat map (Fig 5). The time series included 60 months in total from January 2013 to December 2017. More cases were reported in August and November and fewer cases in September and December across a five-year period. A trough and low cases were found in June of each year with the consistent range of TB cases (lowest to highest) between 50 and 55 cases. In total, the highest TB cases were in 2017 (700 cases), while the lowest TB cases were in 2013 (574 cases). No obvious peak was observed across months in each *mukim* but moderate peak amplitudes were seen in February and December. Furthermore, the empirical peak was found in Batu for each month, with the maximum cases reported in November. In contrast, Setapak and Hulu Kelang showed lower TB cases for each month compared to the other two *mukim*s. Overall, the highest TB cases were in Batu (1466 cases), while the lowest TB cases were in Setapak (527 cases). Setapak presented a consistent range of TB cases (lowest to highest) for each month, which was from 18 to 27 cases.

### ii. Spatial pattern of TB cases

Moran’s I statistic was applied to identify the global pattern of TB cases in Gombak. The global spatial autocorrelation analysis was 0.0005 (*p*<0.05), thus showing a significant spatial correlation in terms of the distribution of TB cases (global Moran’s I>0) over the five-year period. There was no significant temporal variation in the spatial autocorrelation of TB cases. The absolute value of global Moran’s I increased from 2013 to 2016, before decreasing from 2016 to 2017. The significance in the annual global spatial autocorrelation only existed for 2016 and 2017*(p*<0.05) as well as across five-year period, thus clearly indicating the presence of a clustered distribution of TB cases. No global spatial autocorrelation existed in the other three periods *(p*>0.05), thus indicating that TB cases were randomly distributed during those periods. Therefore, the distribution of TB was not strong spatially-correlated in these three years. The pattern of spatial autocorrelation of TB cases from 2013 to 2017 (with the Moran’s I index ranging from 0.0016 to 0.0050) was shown in Table 1.

**Table 1:** Global Morans’ I index for the TB cases in Gombak, 2013 to 2017.

Year	Moran’s I Index	*Z* statistic	*P* value	Pattern
**2013**	0.0016	1.40	0.16	Random
**2014**	0.0022	1.38	0.17	Random
**2015**	0.0029	1.41	0.16	Random
**2016**	0.0050	1.77	<0.05	Clustered
**2017**	0.0041	1.95	<0.05	Clustered
**Average TB cases for five-year period**	0.0005	1.97	<0.05	Clustered

**Fig 6:**
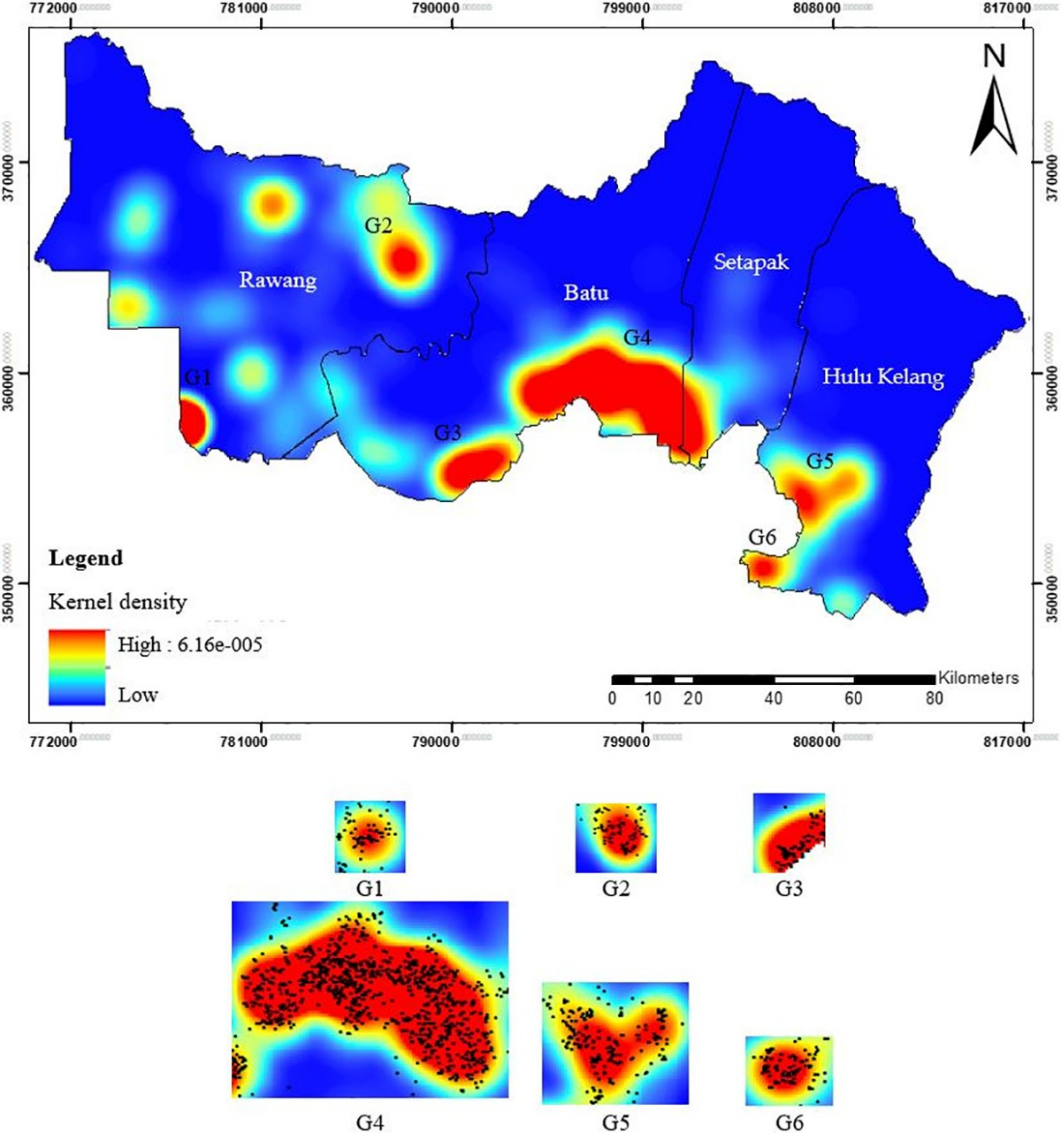
Kernel density map showing the distribution of TB cases in Gombak, 2013 to 2017.

The kernel density map for the distribution of TB cases is illustrated in Fig 6 in which the red and blue shadings display the areas with the highest and lowest densities regions, respectively. The high density localities for the clusters were identified as follows: G1 (Bandar Country Homes; Rawang *mukim*), G2 (Selayang; Rawang *mukim*) and G3 (Kepong; Batu *mukim*), G4 (Desa Tun Razak; Batu and Setapak *mukims*), G5 (Taman Melawati, Hulu Kelang *mukim*), and G6 (Setiawangsa; Hulu Kelang *mukim*). The spatial distribution of TB cases within Gombak was heterogeneous over the five-year period, with the largest cluster of high density region was seen at G4 while the smallest cluster was detected at G6. The ranking from smallest to largest clusters in Gombak was identified as follows: G6 < G5 < G2 < G1 < G3 < G4. The lower density regions of TB cases were predominantly located in the north-west, north-central, and north-east zones.

**Fig 7:**
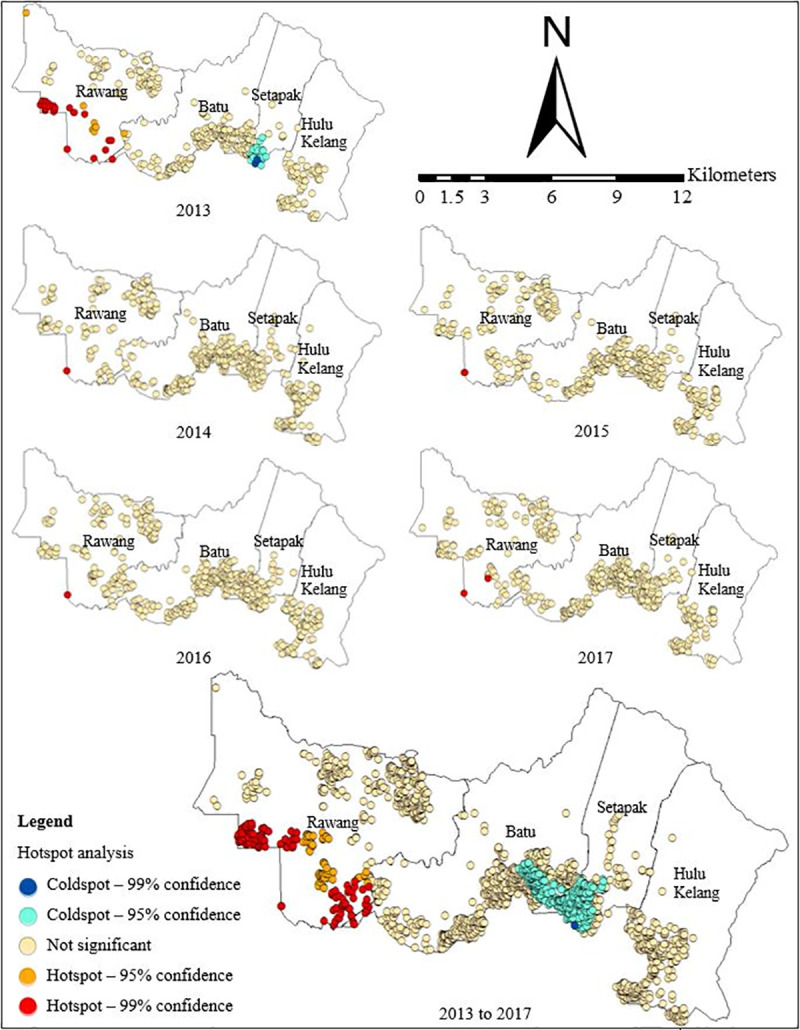
The hotspot analysis of TB cases in Gombak, 2013 to 2017.

Confidence levels of hotspot and coldspot points of TB cases across five-years period using Getis-Ord Gi* are presented in Table 2. The hotspots of TB cases were consistently located in the southwestern part of Gombak at Rawang *mukim* from 2013 to 2017, with 99% (136 points) and 95% (65 points) confidence levels. The location of hotspots did not change much and gradually transferred from the northwestern to southwestern parts of Gombak across the study period. Conversely, coldspots of TB cases were concentrated in the southern part of the district which includes Batu and Setapak *mukim*s, with 95% (378 points) and 99% (1 points) confidence levels. On another note, the location of insignificant clusters of TB cases for each year was present in the northwestern, southern, and southwestern parts of Gombak (1618 points). Temporarily, the hotspots were found in each year while the coldspots were only found in 2013 (Fig 7).

**Table 2:** Summary of hotspot and coldspot points of TB cases in Gombak, 2013 to 2017.

Types of clusters	Confidence levels	Number of hotspot and coldspot points	Residential area / village
Hotspot	99% Confidence	136	Desa Sri Siantan (Sungai Buloh prison), Bandar Tasik Puteri, Desa Puteri, Kampung Gombak, Sungai Pelong, Taman Masquine Sqaure, Taman Amansari Rahman Puteri, and Tiara Puteri
	95% Confidence	65	Bandar Baru Kundang, Kundang Jaya, Kampung Melayu Seri Kundang, Kampung Permata, Taman Tasik Biru, Kampung Setia Kuang, Bukit Ladon, Kampung Baru Cina Kuang, and Taman Sri Putra Mas
Coldspot	95% Confidence	378	Taman Selayang Mutiara, Kampung Laksamana, Kampung Nakhoda, Taman Raintree, Taman Sunaway, Taman Sri Batu Caves, Taman Sunway Batu Caves, Prima Sri Gombak, Taman Samudra, Taman Gombak, Taman Rowther Gombak, Kampung Changkat, Kampung Simpang Tiga, Kampung Tanguit, Taman Kenangan, and Kampung Sungai Chinchin
	99% Confidence	1	Kampung Changkat Permai

### iii. Regression analysis

According to OLS analysis, the explanatory variables are used to explain the risk factor of the TB cases using sociodemographic factor (OLS1), environmental factor (OLS2), and both sociodemographic and environmental factors (OLS3). These models (OLS1, OLS2, and OLS3) met most of the requirements of the OLS method, i.e. the robust probabilities for the explanatory variable coefficients were statistically significant (*p*<0.05); the variance inflation factor (VIF) values were low (VIF<7.5), thus suggesting no multicollinearity are detected among the explanatory variables. The Moran’s I of the residuals for the three models were not significant at 0.05, indicating that the residuals were spatially random. The low value of Moran’s index suggested that the presence of spatial components in these models had eliminated the spatial autocorrelation. In short, there are inclusion of spatial heterogeneity of TB cases. Table 3 shows the summary of the OLS models.

**Table 3:** Summary of OLS models.

Statistics	Models		
	OLS1	OLS2	OLS3
AIC	53053.31	48336.51	48236.52
R^2^	0.06	0.79	0.79
Adjusted R^2^	0.06	0.78	0.79

OLS1 produced these findings: gender, nationality, educational level, employment status, health care worker status, income status residency, and smoking status have non-stationarity influences on the TB cases. This means, these eight variables might be important predictors of the TB cases for some locations in Gombak. No statistically significant autocorrelation was found in the residuals for OLS1 (Moran`s I = 0.006, *Z* = 1.195, *p* = 0.232).

Meanwhile, OLS2 showed these findings: AQI (lags 1–3), CO (lags 2–4), NO_2_ (lags 2 and 3), SO_2_ (lags 1–6), PM_10_ (lags 5 and 6), rainfall (lag 2–6), temperature (lags 1–5), wind speed (lags 4 and 5), relative humidity (lags 2–6), and atmospheric pressure (lag 6) have non-stationarity influences on the TB cases. The analysis of OLS2 shows that air pollution and weather can be important keys of TB cases for some locations in Gombak, such as that described in OLS3. No statistically significant autocorrelation was found in the residuals for OLS2 (Moran`s I = 0.005, *Z* = 0.799, *p* = 0.424).

Additionally, OLS3 presented these findings (*p*<0.01): gender, nationality, residency, employment status, income status, AQI (lags 1–3), CO (lags 2–4), NO_2_ (lags 2 and 3), SO_2_ (lags 1–6), PM_10_ (lags 5 and 6), rainfall (lag 2–6), temperature (lags 1–5), wind speed (lags 4 and 5), relative humidity (lags 2–6), and atmospheric pressure (lag 6) have non-stationarity influences on the TB cases. The findings of OLS3 show that both sociodemographic and environmental factors are important predictors of the TB cases in some locations in Gombak, such as that described in OLS1 and OLS2. No statistically significant autocorrelation was found in the residuals for OLS3 (Moran`s I = 0.003, Z = 0.437, *p* = 0.662).

There was not much difference in the AIC values reported from GWR2 and GWR3; whereby the values of these two models were lower than GWR1. Furthermore, higher R^2^ and adjusted R^2^ values were for GWR2 and GWR3, compared to GWR1. It is shown that the GWR2 and GWR3 performed better than GWR1, but the best is GWR2. Table 4 shows the summary of the GWR models.

**Table 4:** Summary of GWR models.

Statistics	Models		
	GWR1	GWR2	GWR3
Bandwidth	25	34	30
AIC	1357.60	151.79	12.42
R^2^	0.70	0.98	0.90
Adjusted R^2^	0.16	0.95	1.00

**Fig 8:**
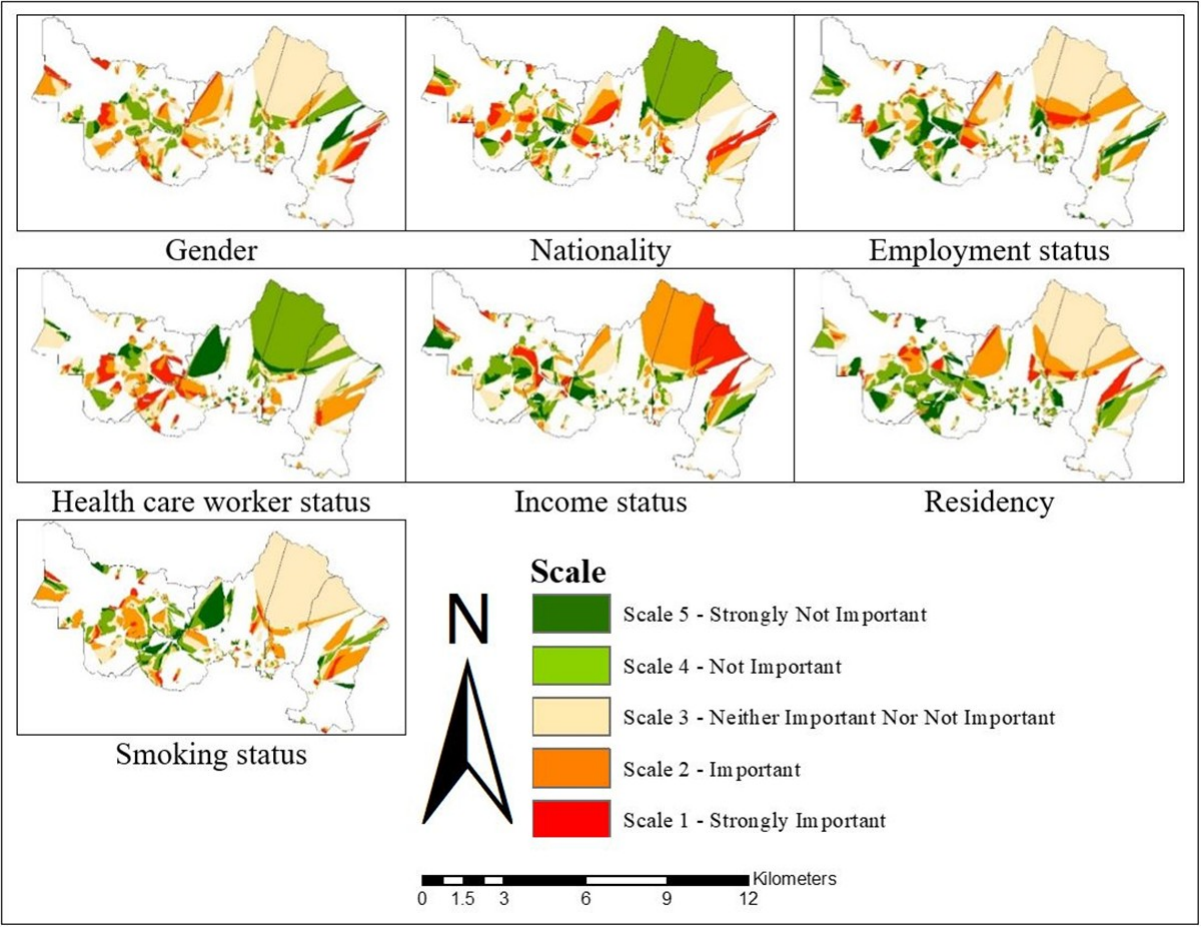
Spatial varying local coefficient estimated for GWR1 in Gombak, 2013 to 2017.

**Fig 9:**
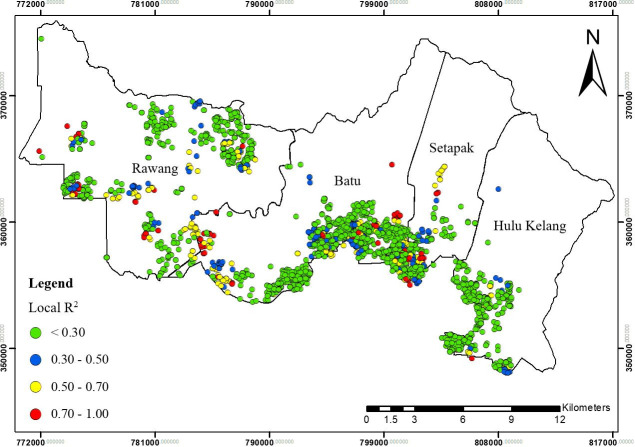
Spatial distribution of local R^2^ for GWR1 in Gombak, 2013 to 2017.

**Fig 10:**
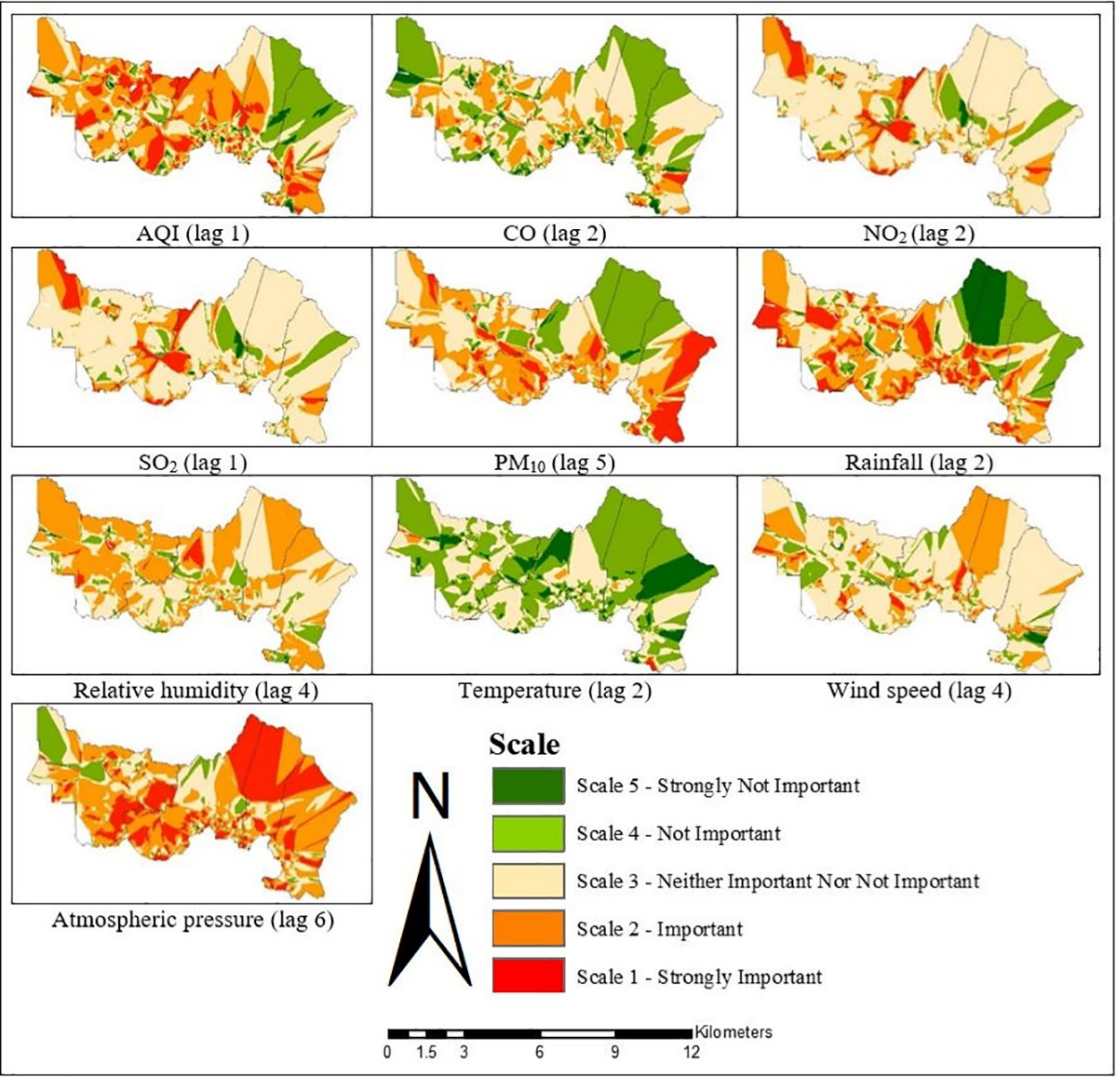
Spatial varying local coefficient estimated for GWR2 in Gombak, 2013 to 2017.

**Fig 11:**
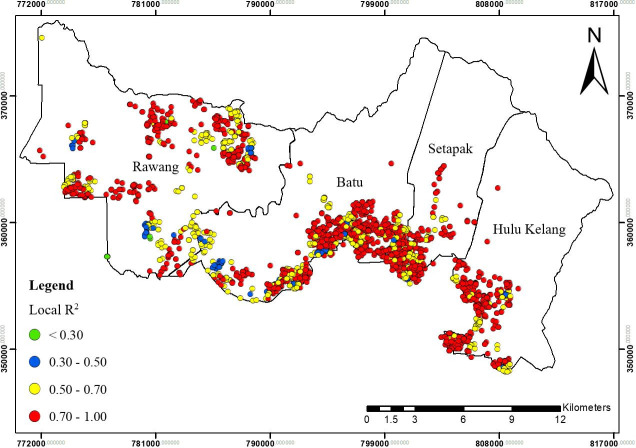
Spatial distribution of local R^2^ for GWR2 in Gombak, 2013 to 2017.

**Fig 12:**
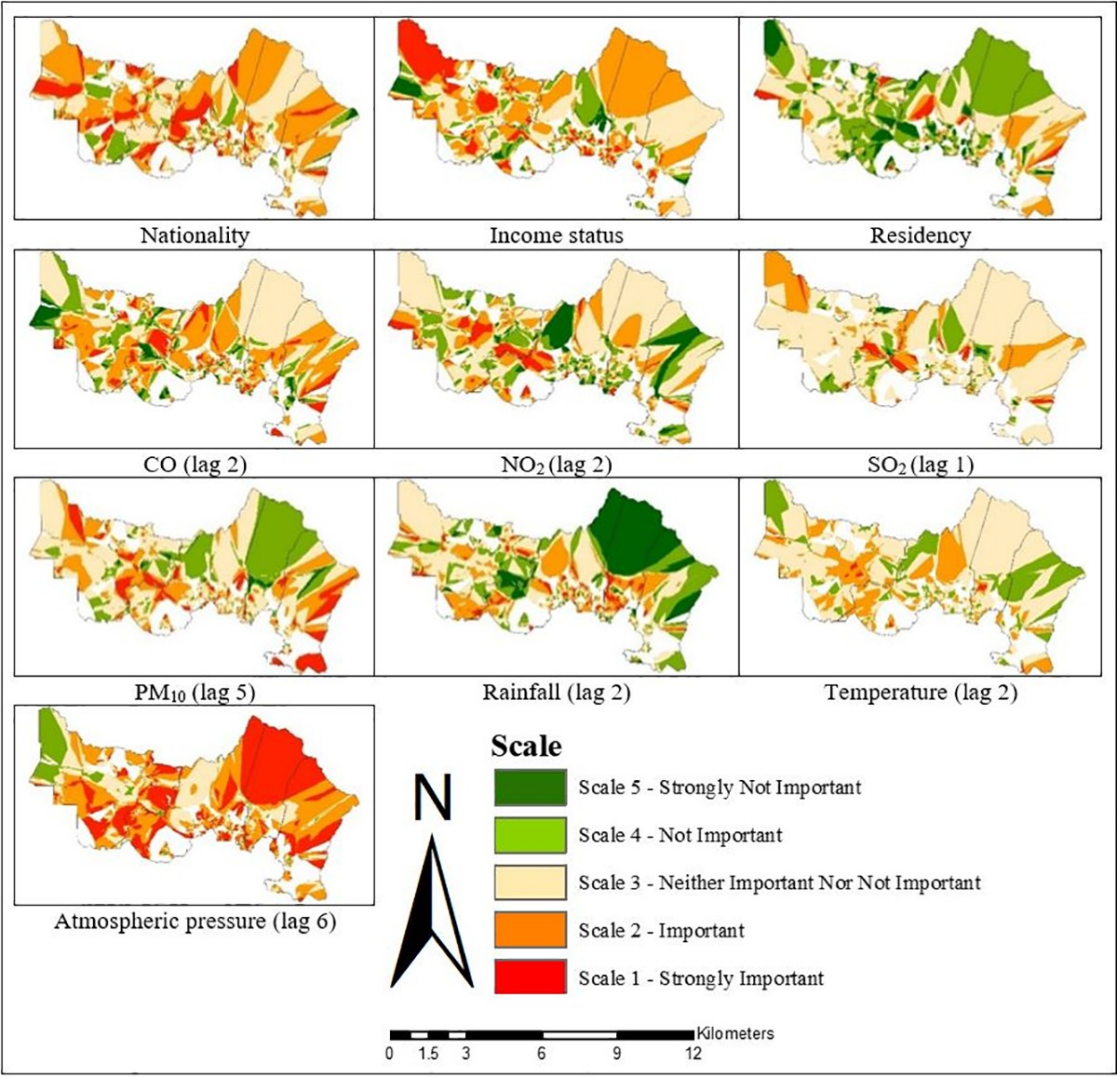
Spatial varying local coefficient estimated for GWR3 in Gombak, 2013 to 2017.

**Fig 13:**
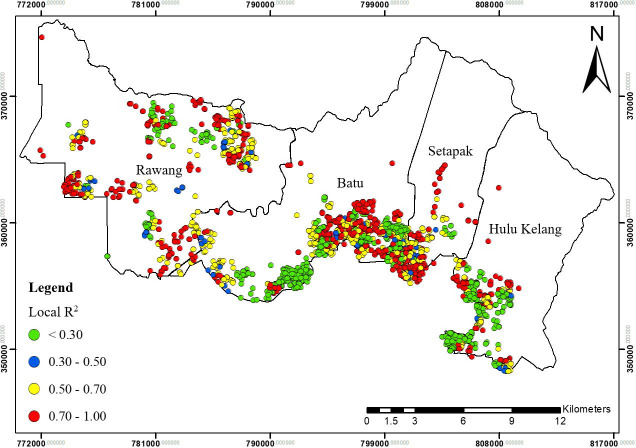
Spatial distribution of local R^2^ for GWR3 in Gombak, 2013 to 2017.

While the OLS identifies the key factors contributing to the TB cases on a global scale model, the GWR helps identify the neighbourhoods where the explanatory variables might be most effective to explain the occurrence of TB cases on a local scale model (Figs 8–13). The visualisation of the local coefficients with different intensity was mapped for each GWR in Figs 8, 10 and 12 to better understand the magnitude and direction of the spatial relationship between each explanatory variable with TB cases. The results clearly demonstrated the existence of an unstable local spatial dependence between TB cases and its risk factors. The five scales of spatial varying local coefficients estimated for GWR1, GWR2, and GWR3: strongly important, important, neither important nor not important, not important, and strongly not important represented the levels of association between each explanatory variable with TB cases ranking from strongest association to weakest association. The highest coefficient was marked by the darkest red colour while the lowest coefficient by the darkest green colour. The higher the coefficient, the stronger the association.

The local R^2^ values further indicated the ability of explanatory variables to explain the spatial variance of TB cases at different locations (Figs 9, 11 and 13). These figures collectively displayed the profound impact of sociodemographic and environmental components on overall TB cases patterns in Gombak. The four classes of spatial varying local R^2^ values for GWR1, GWR2, and GWR3: > 0.70, 0.50–0.70, 0.30–0.50, and < 0.30 represented the levels of association between the combinatory set of explanatory variables with TB cases ranking from “strongest association” to “weakest association”. Among the GWR models, the highest cases of TB for classes of local R^2^ values > 0.70 were observed using the GWR2 model, followed by the GWR3 and GWR1. In contrast, the highest cases of TB for classes of local R^2^ values < 0.30 were found in the GWR1, followed by the GWR3, and the GWR2 (Table 5).

**Table 5:** Total cases of TB classified according to the different classes of local R^2^ values.

	GWR Models		
Local R^2^ values	GWR1	GWR2	GWR3
> 0.70	119	2006	1055
0.50–0.70	254	841	858
0.30–0.50	292	153	154
< 0.30	2560	225	1158

For GWR1 (R^2^ = 0.70), the TB cases were influenced by seven sociodemographic variables. The role of sociodemographic variables was important in certain areas in Gombak (Fig 8) as follows: gender (east-south-east and from north-north west to west, and south-south-west), nationality (east-south-east, centre, north-north-west, and west to west-south-west), employment status (north, centre, and west-south-west), health care worker status (from west to south, and east-south-east), income status (from north-east to east, north-north-west, and west-north-west), residency (from centre to east, north, north-north-west, and west-north-west), and smoking status (east-south-east, centre, and west-north-west). In overall, income status showed the largest areas for the highest spatial varying local coefficient values while health care worker status exhibited the largest areas for the lowest spatial varying local coefficient values for GWR1. The value of local R^2^ varied from 0.00 to 0.93 in the Gombak (Fig 9). The local R^2^ values > 0.70 were mainly concentrated in south-south-east, south, and from south-south-west to west-north-west zones of Gombak which consisted of 119 cases of TB, while the local R^2^ values < 0.30 were observed in most areas of the district which consist of 2560 cases of TB. This suggested the low ability of sociodemographic variables to explain the spatial variation of TB cases across the region compared to GWR2 and GWR3 (Figs 11 and 13).Compared to GWR1 and GWR3, GWR2 explains the better performance of goodness-of-fit (R^2^ = 0.98), in which TB cases were influenced by ten environmental variables. The role of environmental variables was important at the majority of the areas in Gombak (Fig 10) as follows: AQI; lag 1 (most of the areas except north-east), CO; lag 2 (most of the areas except north-east and north-west), NO_2_; lag 2 (from centre to north and parts of north-west), SO_2_; lag 1 (south-east, from north to centre, and north-west), PM_10_; lag 5 (most of the areas except from north to east), rainfall; lag 2 (most of the areas except from north to east), relative humidity; lag 4 (most of the areas except from east to south-east), temperature; lag 2 (few parts in south-east), wind speed; lag 4 (centre, south, and west), and atmospheric pressure; lag 6 (most of the areas except from north-west and north-central). Generally, atmospheric pressure at lag 6 showed the largest areas for the highest spatial varying local coefficient values while temperature at lag 2 exhibited the largest areas for the lowest spatial varying local coefficient values for GWR2. The value of local R^2^ varied from 0.00 to 1.00 in the Gombak (Fig 11). The local R^2^ values > 0.70 were identified in most areas of Gombak which consisted of 2006 cases of TB, while classes of local R^2^ values < 0.30 were scattered in few areas of the district except east and west-south-west zones which consisted of 225 cases of TB; suggesting the high ability of environmental variables to explain the spatial variation of TB cases across the region compared to GWR1 and GWR3 (Figs 9 and 13).

GWR3 (R^2^ = 0.90) showed that TB cases were influenced by three sociodemographic variables and seven environmental variables. The role of sociodemographic and environmental factors was important in most of the areas in Gombak (Fig 12) as follows: nationality (most of the areas except south-west), income status (most of the areas except parts of north-central and west), residency (only few areas in east-south-east, centre and west), CO; lag 2 (most of the areas except north-east, north-west, and few parts in south), NO_2_; lag 2 (most of the areas except from north-east to east, and north-west), SO_2_; lag 1 (from east to south-east and centre to north-west), PM_10_; lag 5 (most of the areas except north-east and parts in south), rainfall; lag 2 (most of the areas except from north-east to east, south-east, and north-west), temperature; lag 2 (most of the areas except from north-east to east, north-west, and few parts in south), and atmospheric pressure; lag 6 (most of the areas except north-west). All in all, atmospheric pressure at lag 6 presented the largest areas for the highest spatial varying local coefficient values while residency exhibited the largest areas for the lowest spatial varying local coefficient values for GWR3. The value of local R^2^ varied from 0.00 to 1.00 in the Gombak (Fig 13). The local R^2^ values > 0.70 were identified in most areas of Gombak except south-west zone which consisted of 1055 cases of TB, while classes of local R^2^ values < 0.30 were presented in most areas of the district except south-west zone which consisted of 1158 cases of TB. The majority of lowest values of local R^2^ indicated the moderate ability of sociodemographic and environmental variables to explain the spatial variation of TB cases across the region compared to GWR1 and GWR2 (Figs 9 and 11).

The hotspot analysis and GWR showed different findings. The hotspot analysis only focused on the total intensity (i.e. the total TB cases) for this study and is a straightforward interpretation of spatial analysis without including any explanatory variables, whereas the GWR interpreted based on the explanatory variables. This study identified the prison as the hotspot area because it has high TB cases, whereby 220 of the patients are inmates and prison workers. On the other hand, there was insignificant difference among them for each sociodemographic and environmental variable category. For example, most of them are male (99.09%; 218 cases), Malaysian (94.09%; 220 cases), unemployed (83.18%; 183 cases), not a health care worker (99.55%; 219 cases), no permanent income (96.36%; 212 cases), and the prison is located in the urban area. Additionally, environmental conditions were similar at the prison area. This is the reason why the GWR findings did not detect any local coefficient raster (Figs 8, 10 and 12) and observed the lowest local R^2^ values (Figs 9, 11 and 13) for the prison area. In contrast, other areas showed that there is variation and spatial relationship among TB patients in each category of the explanatory variables, which is due to obvious differences between them.

## Discussion

This study found spatial heterogeneities of TB cases over the entire areas of Gombak during the study period. The slow rising trend of TB was mainly caused by the increase in the proportion of smear positive TB cases. This was generally consistent with previous research [75, 76]. Researchers should focus on the control of smear positive TB in their implementation plan to control the main source of infection [77] as this increasing trend was not seen in the other TB diagnostic categories. However, patients with smear negative TB also contributed to a quarter (24.23%) of the total TB cases. Worse still, if no treatment was given, half of them may convert into smear positive TB [78]. Therefore, the government has been strengthening measures to improve delays in the diagnosis and notification of smear negative TB cases. The variation in the rate of TB incidence (per 100,000 population) across *mukims* was substantial; some *mukims* reported higher disease incidence than other *mukims*. The expansion of prevention measures could be challenging in high-risk areas.

Temporally, no obvious peak was observed in each year during the study period, possibly indicating that the preventive and control efforts by the Ministry of Health Malaysia were successful. The lack of temporal pattern over the 12-month cycle each year was consistent with the patterns observed in neighbouring countries including Indonesia [34], Thailand [79], and Singapore [80]. As Malaysia was located immediately north of the equator, the weather is hot, humid, and rainy climate throughout the year. The stable temporal pattern of TB cases could be attributed to the local climate and no obvious seasonal pattern was observed as occurred in subtemperate/temperate countries located at the southern and northern hemispheres such as China, Korea, Italy, and Japan.

Spatially speaking, the distribution of TB cases was geographically aggregated. The difference of IR among *mukims* exhibited one of the visions of public health effort in reducing the regional difference of TB distribution towards achieving health equity. The absolute value of global Moran’s I revealed a positive correlation among cases from 2013 to 2017, thus indicating a clustered distribution of TB cases in Gombak. This is probably because each TB case may affect their surrounding areas through population morbidity, have similar dietary habits, living habits, and environmental conditions. These findings are consistent with the previous spatial analysis that showed significant spatial autocorrelation of global TB distribution [81], national TB distribution [82], and the TB distribution in a city [83]. Therefore, it is not surprising that our study also revealed a different trend of TB cases for each year based on the Global Moran’s I. Additionally, the distribution of TB cases in the last two years exhibited a clustered pattern, indicating that each case was located near to each other. In contrast, 2013 to 2015 showed randomly spread of TB which indicated that there was no aggregation of cases across the area. The random effects were probably disturbed by the confounding effects that also captured the presence of spatial heterogeneity in the data [84]. In other words, the findings indicated that the TB cases in Gombak was inconsistently strong spatial cluster. This requires further analysis in future studies because the implementation of control measures will be more difficult for cases with a dispersed and random pattern. Previously, the control measures of TB or other infectious diseases were often implemented without a clear understanding of the distribution pattern, hence the findings of this study were important in filling this knowledge gap.

Generally, the annual pattern of the hotspot areas was the same in the Gombak district with clear spread pattern and trend in relation to the frequency of TB cases. In contrast, the changes in the risk level pattern were also detected, thus suggesting local transmission of the disease around a foci (radially) with waves of concentration diffusing from the hotspot areas. Although areas with a lower density of TB would not cast a big numerical impact on the overall risk, these areas warrant a closer look because they can create a large variation in the TB cases.

In this study, the population (kernel) density per area (m^2^) was positively associated with the risk of TB, thus indicating that a one-unit increase per area would greatly increase the risk of TB, as shown by the spatial analysis of TB cases conducted by Murakami *et* al. [85] and Alves *et* al. [86] in Japan and Brazil, respectively. Six high-risk clusters of high-density regions were found through the kernel density estimation, whereas the rest of the regions presented low-risk areas. While the exact mechanism of the spread of TB needs to be explored further, one of the clusters that indicates high-density of TB cases i.e. G1 was related to prison inmates in the Sungai Buloh prison. Congregate settings are known as the risk factor for high transmission of TB due to overcrowding. Transmission risk is much higher in correctional facilities as it houses high-risk people such as inmates, drug addicts, HIV carriers, and immigrants from countries with high TB burden.

The prisons in Selangor recorded the highest number of infected TB patients compared to other states in Malaysia, besides Penang [87]. Hence, maximizing the distance between inmates’ housing could reduce the TB risk among prison inmates. Nevertheless, the general infrastructures of prisons are usually gloomy, poor lit, and poor ventilation. As a result, prisoners are exposed to many communicable diseases such as TB that it is even likely for the disease to be endemic in prisons. Prison offers a unique setting in the TB treatment modality because it provides a perfect opportunity for patients to complete the treatment. A well-managed prison health care system could ensure inmates completed their TB treatment including those infected with latent TB before being released, may reduce transmission of TB in the community. Furthermore, the largest cluster of high-density regions of TB cases i.e., G4 contributed to population growth and industrialization, which enhanced by rapid urbanization in the border region between Gombak district and Federal Territory of Kuala Lumpur. The low-density regions from the northern to northeastern district are composed of hilly areas whereby most of them covered with forests, and thus a low number of people inhabiting the areas. Moderate risk of TB cases at the west and a part of south is worrying, as it might indicate the failure or delay in diagnosis, making the potential areas for transmission of new TB cases. The ranking of high-density regions from smallest to largest will aid in prioritizing actions for improving the TB surveillance in Gombak.

The spatial pattern of TB cases further depicted the hotspots areas using the Getis- Ord Gi* technique, whereby the statistically significant clusters are essential for resource allocation and the ability to track locations could assist in the interventions to reduce TB burden. Moreover, it is well known that hotspot of TB cases may be common in the states with the highest disease burden [88]. Gombak ranked first among the nine districts in Selangor in terms of TB incidence [3]. The high risk areas were similar each year in which the southwestern region where the prison was located remained the most critical regions for TB cases. This was in agreement with a previous study that identified prisoners as a high-risk population for TB [89]. Through the hotspot analysis, there was a little variation in the distribution of hotspot locations over time because most of them were concentrated in the southwestern and few in the northwestern of Gombak. Therefore, the targeted hotspot areas warrant further investigation to determine the cause, either was it due to failure in TB prevention and control program, or under-detection of cases.

Interestingly, this study found that the significant hotspot areas of TB cases with 95% and 99% confidence levels as shown by the Getis-Ord Gi* technique was located at the similar areas with the high-density region i.e., G1 identified by the kernel density estimation. The kernel density estimation was only able to determine the clusters of high-density regions of TB cases in Gombak while the hotspot analysis could detect the statistical significance of the clusters. Specifically, kernel density estimation detected the highest value at point location, and it diminished as the distance from the point location increased, yet the highest value might not be statistically significant. This study suggested that the kernel density estimation should be used together with the hotspot analysis to detect the high-risk locations of TB cases in Gombak.

This study found that GWR models were a better fit than OLS models. GWR models showed a positive correlation of sociodemographic and environmental factors with TB cases. GWR also illustrated a coefficient raster that helps to observe the variable density in the study area. The reported results are similar to Wang *et* al. [90] that compared between OLS and GWR models to analyse the spatial distribution of TB in mainland China and its association with socioeconomic factors. They also found that GWR was a better fit and performed better in terms of results than OLS. Furthermore, the GWR2 fitted better than GWR1, thus suggesting that environmental factors had a greater influence compared to sociodemographic factors in this study.

Although all the seven sociodemographic variables were identified as the risk factor of TB cases, their impacts were not the same. The income status exhibited the largest areas for strong association with TB cases while the health care worker status showed the largest areas for week association with TB cases. This suggests that health care status do not have a uniform and spatial stationary interaction with TB cases. TB cases were higher among males compared to females, possibly due to a higher risk of TB exposure due to the differences in socioeconomic and lifestyle factors. Furthermore, males regularly smoke tobacco and drink alcohol. These habits can lead to co-morbid conditions [91] and increase the risk of TB infection. A previous study has reported that alcohol and nicotine significantly suppress cellular immune function and production of TNF-α [92]. The gender differences also had a close relationship with use of sex steroids and the immune responses to anti-mycobacterial [93]. Furthermore, occupational risk was an important risk factor of TB transmission as evident by the health care workers who were exposed to the *Mycobacterium tuberculosis* while taking care of the infected patients. We found that the relationship between TB cases and health care worker status was high and strongly important risk area from west to south and southeast areas. An area with higher cases of TB patients will expose health care workers more frequently to the bacilli when these patients seek treatment at health facilities, putting health care workers more at risk of infection. A meta-analysis by Uden *et* al. [94] involving nine studies which was conducted in a mixture of high (*n* = 4) and non-high (*n* = 5) TB burden countries found health care workers had higher risk of active TB infection compared to the general population (pooled IR = 2.94% [95% CI: 1.67–5.19]).

TB cases were high at more developed and urban areas. Urbanization and hope for a better future has resulted in rural-urban migration. Migration of residents into certain areas also resulted in a sharp increase in the local population. Our study showed that the urban patients were engaged in food, transportation, construction, marketing (primary industries), and other industrial workforces. People involved in primary industries mostly have low incomes, poor living conditions, and insufficient knowledge of TB. All these would increase the transmission of TB. TB is known to be a poverty-related disease, hence income and employment status should be taken into consideration in decision-making related to public health policies. Moreover, this study found that household income could be a protective factor against TB in some urban areas. This is in contrast with the fact that low socioeconomic status had a higher tendency to cause TB [95, 96]. Total household income could be the reason behind this finding. Therefore, future studies should investigate the actual household income rather than just the income status i.e. permanent and not permanent incomes. In addition, the GWR results showed that this protection was stronger in urban areas in the central, southern, and western regions. The proportion of nationality was also a strong predictor of TB. Non-Malaysians had a lower socioeconomic status. Furthermore, the overcrowded and poor living environment among foreigners could increase the risk of developing TB.

One interesting finding in this study is that the two major pollutants such as smoke (NO_2_, CO, and SO_2_) and PM_10_ were associated with TB cases. A cohort study with a median follow-up of seven years in Taiwan found that the risk of active TB was 1.33 (1.04–1.70) with a 10 ppb increase in NO_2_, 1.89 (0.78–4.58) with a 10 ppm increase in CO, and 0.95 (0.78–1.17) with a 10 μg/m^3^ increase in PM_10_, [97], whereas NO_2_ could increase the risk of TB by 5.4 times more than PM_10_ [98]. Huang [99] showed that 10 *μ*g/m^3^ in SO_2_ could increase up to 4.6% of pulmonary TB incidence rate. These exogenous pollutants are easily dissolved in water; thus, allowing them to easily corrode the mucous membrane lining. Inhalation of these pollutants can subsequently produce irritation and inflammation of the airways on the upper respiratory tract [100]. These pollutant matter may weaken the body’s immune system and increase the risk of TB infections through (i) reduction of tumour necrosis factor-α (TNF-α) and interferon-gamma production (IFN-g) in peripheral blood monocytes [101, 102]; (ii) inhibition of serum neutralizing antibodies formation [103]; (iii) alterations in alveolar macrophage phagocytosis and blood mononuclear cells [25]; and (d) increased susceptibility to infection via upregulation of receptors involved in pathogen invasion [104].

The atmospheric pressure exhibited the largest areas for strong association with TB cases while the temperature status showed the largest areas for week association with TB cases. Temperature could be responsible for this uneven distribution of spatial association with TB cases. Previous studies pointed to an association between weather (rainfall, relative humidity, temperature, wind speed, and atmospheric pressure) and TB occurrence. In countries with hot and humid weather, high intensity of rainfall may provide a conducive environment for the *Mycobacterium tuberculosis* to grow and reproduce more rapidly [105]. Beiranvand *et* al. [106] concluded that rainfall is one of the direct indicators of humidity that influenced the TB incidence rate in Iran. The higher the humidity, the slower the air circulation, furnishing conditions for *Mycobacterium tuberculosis* to be attached to ambient particulates and stay in the air for a longer time [107]. In addition, high humidity can facilitate the formation of larger aerosols composed of the tubercle bacillus; thus, increasing the infectious bacterial dose entering the body, overcoming the immune system, and spreading the disease [108]. High temperature may restrain the development of TB by affecting blood pressure, aspiration, and biological vitality to increase the infection rate [109]. Furthermore, the recombinant strain of *Mycobacterium tuberculosis* can stop growing and even could be destroyed when temperature exceeds 37°C [110]. Higher speed of wind can exacerbate TB infection by increasing a person’s susceptibility to extreme weather and accelerate the air ventilation, but lower speed of wind can enhance the spread of *Mycobacterium tuberculosis* floating in the wind [107]. High air pressure enhances atmospheric flow, promoting the transmission of *Mycobacterium tuberculosis* [111]. The majority of the risk factors had a strong impact on the north-west, north-central, and east regions. In view of this, future efforts in the control and prevention of TB should be concentrated in these high-risk areas.

Despite the multiple findings from this study, there are a few limitations to this study. First of all, the analysis was performed at the district level. To strengthen the findings, it might be desirable to examine the associated factors of TB cases at lower geographical units such as cities and counties. Secondly, certain risk factors were not well studied due to the unavailability of data. Further research should widen the investigation of the risk factors of TB cases by including sociodemographic variables (population density, the proportion of homeless population), economic factors (per capita of gross domestic product, total household income), and health-related factors (death rate, numbers of beds/doctors). However, the majority of the variables were available only at the *mukim* level and not at the city or country levels. It is not statistically appropriate to conduct regression analysis for many factors with such a small sample size (four *mukims*). Therefore, the analysis was done for the sociodemographic and environmental variables with data available at the district level, and 17 important factors were identified. These factors should be taken into consideration in future studies as they can help to formulate policies related to TB intervention. Thirdly, this analysis utilised the monthly TB data of four *mukims* in Gombak, but studies with higher temporal patterns on a daily or weekly basis and a longer period of 10 years would provide more precise information. It can also reduce any misleading or biased outputs. Lastly, there can potentially be under-reporting of cases among people who sought care or were diagnosed in private health care services.

## Conclusions

This study identified the spatial distribution of TB cases in Gombak from January 2013 to December 2017. The cases were higher during August and November each year, with a slowly rising trend and no obvious peak over the five years. TB cases also showed a positive global spatial autocorrelation in 2016 and 2017. The high-high clustering areas of TB cases mainly concentrated in the south-west region where the Sungai Buloh prison is located. The geospatial non-stationary analysis suggested that GWR2 was the best model to determine the distribution of TB cases with the highest R^2^ i.e. 0.98. This study also found that sociodemographic factors such as gender, nationality, employment status, health care worker status, income status, residency, and smoking status; and environmental factors such as AQI (lag 1), CO (lag 2), NO_2_ (lag 2), SO_2_ (lag 1), PM_10_ (lag 5), rainfall (lag 2), relative humidity (lag 4), temperature (lag 2), wind speed (lag 4), and atmospheric pressure (lag 6) played an important role that affected TB cases in varying degrees and different areas in Gombak. The strongest association between income status and atmospheric pressure at lag 6 with TB cases was found in this study, suggesting that these two factors could be the key controlling variables that determine the overall casualties caused by TB cases in Gombak. Therefore, the Ministry of Health Malaysia should give more priorities towards TB control in these areas. This study provided a better understanding of the spatial approach in epidemiology. It also highlighted prison as an important environmental factor for prospective TB surveillance.

## Supporting information

S1 Data (XLSX)

S2 Data (XLSX)

S3 Data (XLSX)

S4 Data (XLSX)

## Abbreviations

ACB: acid fast bacilli
AQI: air quality index
CO: carbon monoxide
GWR: geographically weight regression
IR: incidence rate
NO_2_: nitrogen dioxide
OLS: ordinary least squares
PM_10_: particulate matter 10
SO_2_: sulphur dioxide
TB: tuberculosis
WHO: World Health Organization
